# Improving care for mental health, neurological and substance use conditions by enhancing pre‐service education for doctors and nurses: a new WHO guide

**DOI:** 10.1002/wps.21322

**Published:** 2025-05-15

**Authors:** Sherianne Kramer, Brandon Gray, Shekhar Saxena, Sian Lewis, Ricardo León‐Bórquez, Mark van Ommeren

**Affiliations:** ^1^ Department of Mental Health, Brain Health and Substance Use World Health Organization Geneva Switzerland; ^2^ Harvard T.H. Chan School of Public Health Boston MA USA; ^3^ World Federation for Medical Education

Recent efforts to reduce the enormous treatment gap and significant mental health care workforce shortages have focused on training primary care staff to recognize and provide care for people with mental health, neurological and substance use (MNS) conditions^1^. In‐service (or on‐the‐job) training is a useful and evidence‐based approach for upskilling doctors and nurses to manage these conditions^2^. However, an additional and more sustainable approach is to strengthen competency‐based mental health care education for health care providers before they enter the workforce^3^. Here we argue for a stronger emphasis on pre‐service university education for doctors and nurses in the areas of mental health, brain health and substance use, and outline the work that the World Health Organization (WHO) is undertaking to promote this approach.

Both pre‐service education and in‐service training are necessary to secure a competent workforce to provide care for people with MNS conditions. Pre‐service education equips health care providers with basic professional attitudes, knowledge and skills during their undergraduate or first‐degree training. It sets the foundations for clinical practice and provides an important reference point for follow‐up in‐service, post‐graduate and continuous education.

Strengthening the focus on MNS care in the pre‐service education of doctors and nurses is especially valuable because it ensures that primary health care providers accept responsibility for MNS care from the earliest stages in their careers^4^. Universities can, through relevant assessment, ensure the competence of their graduates before they enter the workforce. And, because new graduates will be better equipped to provide care for people with MNS conditions, there will be a reduced need to rely on in‐service training^5^. Importantly, good‐quality pre‐service education can help to reduce stigma related to MNS conditions among health care providers^6^.

Nonetheless, pre‐service education in MNS care is currently often inadequate, despite its strategic value. Commonly cited obstacles to embedding MNS care into pre‐service education for medical and nursing students include time constraints, too few trained educators and clinical sites available, lack of endorsement from decision‐makers, and faculty staff resistance due to increased workload or territorialism^3^.

The pre‐service education on providing care for people with MNS conditions that is available for medical and nursing undergraduates varies widely in quality. It is typically brief and theoretical, and so insufficiently geared to the tasks that doctors and nurses can be expected to do^7^. For example, students may learn about prescribing psychotropic medicines, but may be insufficiently trained in promoting human rights and providing non‐pharmacological management and support (e.g., psychoeducation, stress management, psychosocial support). Too often, clinical internships are placed in specialized care settings, such as psychiatric hospitals, which do not adequately reflect the MNS needs that medical or nursing students are likely to encounter after graduation.

The result is that medical and nursing graduates regularly fail to achieve the competencies they need to adequately identify, support and refer people experiencing emotional distress, MNS conditions and social difficulties. Common conditions such as depression, anxiety and substance use disorders go unnoticed or are poorly managed by primary health care providers. Person‐centred, rights‐based, recovery‐oriented care is rare.

There is an urgent need to advance competency‐based pre‐service education programmes to equip future doctors and nurses in preventing, promoting, and providing care for people with MNS conditions, and so better prepare them for any subsequent postgraduate education and in‐service work. This will in turn improve the extent and quality of care for people living with MNS conditions globally.

The WHO's Mental Health Gap Action Programme (mhGAP) provides evidence‐based guidance and tools for integrating priority MNS conditions into general health care settings. While it is most often used as a clinical tool or as the basis for in‐service training, we encourage educators and decision‐makers to also use the mhGAP Intervention Guide^8^ as a key tool for enhancing pre‐service education^4^. Experience from around the world shows that mhGAP‐based pre‐service training can be implemented in diverse settings and programmes^3^.

To advance the necessary reform to pre‐service education, WHO has worked to identify the core competencies that doctors and nurses need to provide quality MNS care, and how these can be embedded in medical and nursing undergraduate curricula. In partnership with other international organizations, we have published a practical guide for health care workforce decision‐makers and educators^9^. Using a competency‐based approach that is associated with better learner engagement and preparedness for practice, the guide provides an overview of the key activities and considerations needed to integrate mental health care competencies into a university's teaching processes for professionals non‐specialized in providing care for MNS conditions.

The new guide defines each core competency – and the attitudes, knowledge and skills required to achieve it – that doctors and nurses working in general health care need to support people experiencing MNS conditions. It provides examples of the learning content, teaching methods and assessments to include in undergraduate curricula. And it offers guidance to further support programme implementation, for example on training educators, securing the support and endorsement of faculty, administrators, accreditors and regulatory bodies, and monitoring and evaluation.

Moving forward, the WHO aims to mobilize action to apply this guide and support countries to scale up pre‐service education for MNS care globally. We encourage all stakeholders to participate in this process by advocating for MNS care competencies to be embedded in medical and nursing education and actively working to enable curricular change and sustainable workforce development.
